# Expression and clinical significance of FXYD3 in endometrial cancer

**DOI:** 10.3892/ol.2014.2170

**Published:** 2014-05-23

**Authors:** YIFEI LI, XIA ZHANG, SHUWEN XU, JING GE, JING LIU, LIN LI, GUIYING FANG, YALI MENG, HONGZHEN ZHANG, XIAOFENG SUN

**Affiliations:** 1 Department of Obstetrics and Gynecology, First Hospital of Hebei Medical University, Shijiazhuang, Hebei, P.R. China; 2 Department of Oncology, Institute of Clinical and Experimental Medicine, Country Council of Östergötland, University of Linköping, Linköping 58185, Sweden

**Keywords:** FXYD3, endometrial hyperplasia, endometrial neoplasm, immunohistochemistry

## Abstract

FXYD3 expression is upregulated in numerous cancer cell types. The present study compared the FXDY3 expression in normal endometrium, premalignant lesion and endometrial cancer tissue samples, and investigated the correlation between FXDY3 expression and clinicopathological features. FXYD3 expression was analyzed by streptavidin-peroxidase immunohistochemistry in 21 normal endometrial tissue samples, 18 atypical endometrial hyperplasia samples and 50 tissues obtained from patients diagnosed with endometrial cancer. The percentage of FXYD3-positive cell expression in the normal endometrium, atypical hyperplasia and endometrial cancer tissues samples was 0, 22, and 26%, respectively. The differences between the atypical hyperplasia and endometrial cancer groups were statistically significant when compared with the normal group (P=0.007 and P=0.037, respectively). There was no significant difference between the atypical hyperplasia and endometrial cancer groups. The percentage of FXYD3-positive cells correlated with the fertility frequency (P<0.05). In conclusion, FXYD3 is a potential biomarker for endometrial cancer, and its upregulation may be an early event in endometrial carcinoma progression. In addition, FXYD3 expression in endometrial carcinoma correlates with fertility frequency.

## Introduction

Members of the highly-conserved FXYD family are differentially expressed in a wide variety of mammalian tissues and cancer types (1,2). To date, the family comprises 12 water-insoluble, transmembrane proteins that serve as ion channels and/or ion channel regulators (3–6). All FXYD genes are expressed in early embryonic cells, and the expression of certain FXYD proteins is tissue-specific in mammals. FXYD1 is expressed in skeletal muscle and the myocardium, FXYD2 is primarily expressed in kidney epithelial basement membranes, the bile duct and in cholangiocarcinoma cells, FXYD3 is primarily expressed in the liver, pancreas, stomach, colon, prostate, lung, kidney, skeletal muscle and epidermal cells, FXYD4 is primarily expressed in the kidney and distal colon, and FXYD5 is expressed in the brain (7).

Certain FXYD proteins display altered expression in cancer cells. For instance, FXYD2 is differentially expressed in cholangiocarcinoma cells, as is FXYD5 in epithelioid sarcoma, head and neck squamous cell carcinoma, small cell carcinoma, pancreatic cancer cells and breast cancer cells. FXYD3 expression is upregulated in breast cancer tissues and cancer cell lines, intrahepatic cholangiocarcinoma, thyroid cancer, colon cancer, certain prostate cancer cells and in urothelial cancers (8,9). It has also been reported that FXYD3 expression is downregulated in specific prostate cancer cells (10). FXYD proteins have garnered a high level of research focus in recent years, as they appear to play significant physiological and pathophysiological roles in human biology.

As such, FXYD3 is being scrutinized as a potential novel biomarker for cancer (11). The human FXYD3 gene is located on chromosome 19q13.11-q13.12. This gene is 8,428 base pairs long, and is comprised of 9 exons and 8 introns. FXYD3 belongs to the FXYD protein family. It interacts with, and regulates the Na^+^/K^+^-ATPase enzyme, but also acts independently as a chloride ion channel or chloride channel regulator (12).

To the best of our knowledge, FXYD3 expression has not been investigated in association with endometrial cancer. Endometrial cancer is the most common gynecological malignancy. Each year, 142,000 females are diagnosed, and 42,000 females die from this disease worldwide. In the present study, immunohistochemistry was used to detect the differential FXYD3 expression and corresponding pathological changes in endometrial tissue samples obtained from patients diagnosed with endometrial cancer. The correlation between endometrial cancer risk factors, clinicopathological features and FXYD3 expression is analyzed and discussed.

## Materials and methods

### Patients

For immunohistochemistry, formalin-fixed paraffin-embedded tissue blocks were obtained from 50 patients with endometrial cancer and integral clinical data at the First Hospital of Hebei Medical University (Shijiazhuang, Hebei, China) between 2005 and 2007. The patients were diagnosed according to the International Federation of Gynecology Obstetrics (FIGO) Surgical Staging System for Endometrial Cancer (2000) (13). The study also included 18 atypical endometrial hyperplasia and 21 normal endometrium samples. The median age of the patients was 36, 40.5 and 57 years old (range, 22 to 60, 26 to 77, and 33 to 75 years old) for the normal endometrium, atypical endometrial hyperplasia and endometrial cancer groups, respectively. The study was approved by the Ethical Committee at the First Hospital of Hebei Medical University. Patients provided written informed consent.

### Immunohistochemistry

The preparation, specificity and reliability of the rabbit polyclonal FXYD3 antibody used in the study have been described previously (14). Continuous 5 μm sections from paraffin-embedded tissue were deparaffinized, hydrated and rinsed in distilled H_2_O. In order to expose masked epitopes, the sections were boiled in citrate buffer (pH 9.0) in a high pressure cooker for 20 min, and then kept at room temperature for 30 min, followed by a phosphate-buffered saline (PBS; pH 7.4) wash. The activity of endogenous peroxidase was blocked in 3% H_2_O_2_ in methanol for 10 min, and then the sections were washed 3 times in PBS. Subsequent to being blocked with 1.5% horse serum in PBS for 10 min, the sections were incubated with the primary mouse anti-human monoclonal anti-FXYD3 antibody (kindly obtained from Professor Hanswalter Zentgraf, Department of Applied Tumor Virology, University of Heidelberg, Heidelberg, Germany) in 1:2 diluted in PBS (pH 7.4) at 4°C overnight. Next, a biotinlated anti-rabbit Immunoglobulin G antibody (Fuzhou Maixin Biology Technology, Fuzhou, China) was applied for 30 min, followed by incubation of an avidin-biotin-peroxidase complex (Beijing Zhongshan Biology Technology, Beijing, China) for 30 min. The sections were rinsed in PBS between the incubations. The peroxidase reactions were developed using diaminobenzidine (Beijing Zhongshan Biology Technology) for 8 min. Following counterstaining with hematoxylin, the sections were dehydrated and mounted. The breast cancer sections known to be FXYD3-positive were included as positive or negative controls. A negative control was designed for every staining procedure, i.e., PBS instead of the primary antibody.

### Histological analyses

The stained sections were microscopically examined and scored independently by two pathologists who were blinded to the experimental conditions. Yellow-stained granules observed in the cytoplasm and/or the membrane of glandular epithelial cells in the normal endometrium, atypical endometrial hyperplasia and endometrial cancer tumor cells were considered FXYD3-positive cells. A total of 10 different high power fields (10×40) were randomly selected for each sample, and the total number of cells and FXYD3-positive cells were counted. The positive cell rate was calculated as the following: Positive cell rate = ∑positive cells/∑cells × 100. The staining intensity was graded on a scale of 0–3 based on the following criteria: 0 for negative cells or those with no staining, 1 for yellow-stained cells, 2 for orange-stained cells and 3 for brown-stained cells. The percentage of stained cells was classified according to the following system: 0 for ≤5% staining, 1 for 6–25%, 2 for 26–50% and 3 for >50%. The final score was defined as the sum of the staining intensity and the percentage of stained cells in each section, and sections scored from 0 to 6 points. To avoid staining artifacts, the cells in areas with necrosis, poor morphology and section margins were not counted.

### Statistical analyses

For statistical analyses, staining scores of 0 to 3 points were counted as negative and ≥4 points was counted as positive. All data were analyzed using SPSS 13.0 software (SPSS, Inc., Chicago, IL, USA). The χ^2^ method and the Fisher’s exact test were used to examine the correlation between FXYD3 expression in the normal endometrium, atypical endometrial hyperplasia and endometrial carcinoma groups, and the correlation between FXYD3 expression in cancer and clinicopathological variables. All P-values were cited as two-sided, and P<0.05 was considered to indicate a statistically significant difference.

## Results

### FXYD3 expression in normal endometrium, atypical endometrial hyperplasia and endometrial cancer

**Figure 1:**
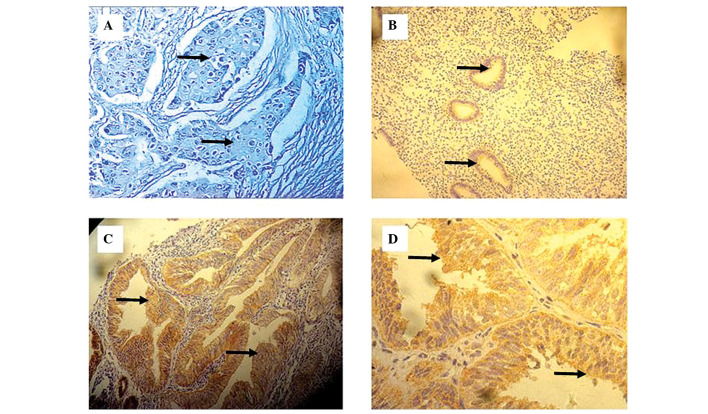
(A) Negative control (a breast cancer sample positive for FXYD3) where the primary FXYD3 was replaced by phosphate-buffered saline showed no staining for FXYD3 in tumor cells (arrow). (B) FXYD3-negative expression in epithelial cells of the normal endometrium. (arrow). (C) Moderate expression of FXYD3 in the epithelial cells of atypical endometrial hyperplasia (arrow). (D) Strong expression of FXYD3 in the tumor cells of the endometrial cancer (arrow).

FXYD3 expression was examined in the normal endometrium samples (n=21), the atypical endometrial hyperplasia samples (n=18) and the endometrial cancer tissue samples from surgically removed specimens (n=50). FXYD3 expression in the cytoplasm and/or normal epithelial membranes and tumor cells, and the staining in the cytoplasm and/or the membrane was heterogeneous and granulous. Among the 50 endometrial cancer tissue samples, 13 exhibited FXYD3-positive cells. However, FXYD3 expression in these samples was heterogeneous, displaying great variation in the numbers of FXYD3-positive cells and the staining intensity in different regions of the same section (Fig. 1).

The percentage of FXYD3-positive cells in the normal endometrium, atypical endometrial hyperplasia and endometrial cancer tissue samples was 0, 22 and 26%, respectively (Table I). The percentage of FXYD3-positive cells in the atypical hyperplasia and endometrial cancer tissues were significantly increased when compared with samples in the normal endometrium group (P=0.007 and P=0.037, respectively). However, there was no significant difference between the atypical hyperplasia and endometrial cancer groups (P=1.000).

### Correlation between FXYD3 expression in endometrial cancer and clinicopathological features

The correlation between FXYD3 expression and different clinicopathological features was examined. Table II shows the correlation between FXYD3 expression and patient age, fertility frequency, blood pressure, plasma sugar and lipid levels, family history of cancer, age of menopause onset, FIGO stage, histopathological type, histological grade, myometrial invasion, cervical involvement, lymph nodal metastases and growth pattern. FXYD3 expression in the endometrial carcinoma group was negatively correlated with fertility frequency. A high fertility frequency corresponded with lower FXYD3 expression (P=0.024).

## Discussion

The present study investigated the correlation between FXYD3 expression and endometrial cancer using immunohistochemical analyses of normal endometrium, atypical hyperplasia and endometrial cancer tissue samples. The correlation between differential FXYD3 expression and several different clinicopathological features was also analyzed. FXYD3 expression in different human tissues has been extensively studied using various methods. FXYD3 is expressed in normal human tissues, including the liver, colon, prostate, lung, pancreas and brain and epithelium. In addition, a growing body of evidence indicates that FXYD3 expression is upregulated in numerous different tumor tissues and tumor cell lines. Moreover, certain studies indicate that tumor malignancy is positively correlated with FXYD3 expression (15–18).

For example, Morrison *et al* used quantitative (q)PCR and northern blotting to demonstrate that FXYD3 was expressed at a significantly higher level in the primary breast cancer tissues obtained from 16 patients, and in eight different human breast cancer cell lines (15). Notably, studies investigating FXYD3 expression in prostate tissues have yielded conflicting results. Grzmil *et al* found that FXYD3 was highly expressed in prostate cancer tissue samples when using cDNA chip technology and qPCR (10). In the same study, the suppression of FXYD3 expression caused a significant decrease in the cellular proliferation of prostate cancer cell lines.

Studies on pancreatic cancer show that FXYD3 expression in cancerous tissues and pancreatic cancer cell lines is significantly higher than in normal pancreatic tissues (16) and in chronic pancreatitis (16–18). In non-small cell lung cancer, FXYD3 expression in tumors for patients with poor prognoses is higher than in those with better prognoses. This indicates that FXYD3 could be an important prognostic secondary indicator (19).

To the best of our knowledge, the present study is the first to examine FXYD3 expression in endometrial cancer tissues. FXYD3 expression was analyzed and compared in tissue sample sections by immunohistochemistry using grading scales that quantified the number of FXYD3-positive cells and the staining intensity of these cells. The percentage of FXYD3-positive cells in the normal endometrium, endometrial hyperplasia and endometrial cancer tissue samples was 0, 22, and 26%, respectively. These results indicate that FXYD3 is expressed in the early stages of endometrial carcinoma formation, suggesting that the upregulation of FXYD3 may be an early event in the progression of endometrial cancer. From these study results, we propose that FXYD3 may be a promising biomarker for endometrial cancer.

The female reproductive system is the target organ for the sex hormones, estrogen and progesterone. Each hormone mediates multiple effects via their specific receptors. Estrogen promotes endometrial cell hyperplasia and vascular proliferation, and induces estrogen receptor and progesterone receptor expression. Progesterone stimulates endometrial cell differentiation and promotes apoptosis in atypical hyperplasia endometrial cells, thus inhibiting excessive growth or transformation (20).

Endometrial cancer progression is correlated with endometrial hyperplasia, elevated estrogen levels and decreased progesterone levels (21). Studies have shown that large doses of estrogen replacement therapy increase the risk of endometrial cancer 2–10-fold (22). Obesity, hypertension and diabetes are three other factors associated with endometrial cancer. The risk of endometrial cancer in diabetic patients or patients with impaired glucose tolerance is 2.8 times greater than that of healthy individuals (23). The present data indicated that FXYD3 expression in endometrial cancer tissues was not significantly correlated with the patient age, blood pressure, menopause onset, plasma glucose and lipid levels, family history of cancer, myometrial invasion, cervical invasion, lymphatic metastasis, clinical cancer stage, growth pattern and histological type of the endometrial cancer tumor (P>0.05). However, a correlation was detected between FXYD3 expression and fertility. This data indicates that lifelong infertility is a risk factor for endometrial cancer. We hypothesize that the effects of estrogen on endometrial tissues are uncontrolled in individuals lacking sufficient amounts of progesterone. During pregnancy, progesterone inhibits menstruation (24). The cell damage, repair, and injury responses in endometrial epithelial cells shut down, and the risk of developing endometrial cancer during pregnancy is reduced. The present study found that females who have never been pregnant are twice as likely to develop endometrial cancer than those who have given birth once. This is particularly true for females who are unable to become pregnant due to failed ovulation and insufficient progesterone levels. This results in endometrial hyperplasia that could progress to endometrial cancer. Our results show that FXYD3 expression in endometrial cancer tissues is correlated with fertility frequency (P=0.024). The risk of developing endometrial carcinoma appears to be higher in females who have never become pregnant when compared with those who have given birth. With each birth, the risk of developing endometrial carcinoma decreases. Whether this correlation is due to progesterone-regulated levels of FXD3 levels or vice versa is unclear. To address this question, future studies examining the correlation between estrogen, progesterone and FXYD3 expression in normal and endometrial cancer cells are required.

## Figures and Tables

**Table I:** FXYD3 expression in normal endometrium, atypical endometrial hyperplasia and endometrial cancer.

		FXYD3 expression, n (%)		
			
Groups	n	Positive	Negative	P-value
Normal endometrium	21	0 (0)	21 (100)	0.037^a^
Atypical hyperplasia	18	4 (22)	14 (78)	1.000^b^
Endometrial cancer	50	13 (26)	37 (74)	0.007^c^

a Atypical hyperplasia vs. normal endometrium;

b Atypical hyperplasia vs. endometrial cancer;

c Endometrial cancer vs. normal endometrium.

**Table II:** FXYD3 expression in the endometrial cancer tissue samples, and clinicopathological features.

		FXYD expression, n (%)		
			
Variables	n	Negative	Positive	P-value
Age, years				0.990
<55	23	17 (74)	6 (26)	
≥55	27	20 (74)	7 (26)	
Births				0.024
None	5	4 (80)	1 (20)	
1	7	2 (29)	5 (71)	
≥2	37	30 (81)	7 (19)	
Blood pressure, mmHg				0.747
<140/90	25	18 (72)	7 (28)	
≥140/90	25	19 (76)	6 (24)	
Plasma glucose, mmol/l				0.586
<6.1	27	19 (70)	8 (30)	
≥6.1	22	17 (77)	5 (23)	
Plasma lipids				0.405
Normal	13	8 (62)	5 (38)	
High	10	8 (80)	2 (20)	
Family history of cancer				1.000
No	43	31 (72)	12 (28)	
Yes	6	5 (83)	1 (17)	
Menopause onset age, years				0.794
<49	21	17 (81)	4 (19)	
49–52	15	10 (67)	5 (33)	
≥52	14	10 (71)	4 (29)	
FIGO stage				0.919
I	33	25 (76)	8 (24)	
II	10	8 (80)	2 (20)	
III	7	5 (71)	2 (29)	
IV	0	0 (0)	0 (0)	
Histopathological type				0.549
Adenocarcinoma	48	35 (73)	13 (27)	
Undifferentiated carcinoma	1	1 (100)	0 (0)	
Small cell carcinoma	1	1 (100)	0 (0)	
Histological grade				1.000
I	0	0 (0)	0 (0)	
II	25	19 (76)	6 (24)	
III	5	4 (80)	1 (20)	
Myometrial invasion				0.372
No	3	3 (100)	0 (0)	
Superficial myometrial invasion	33	24 (73)	9 (27)	
Deep myometrial invasion	13	9 (69)	4 (31)	
Cervical involvement				0.727
No	34	24 (71)	10 (29)	
Yes	15	12 (80)	3 (20)	
Lymph nodal metastases				0.556
No	36	26 (72)	10 (28)	
Yes	3	3 (100)	0 (0)	
Growth pattern				0.682
Limitations	24	17 (71)	7 (29)	
Diffusibility	25	19 (76)	6 (24)	

FIGO, International Federation of Gynecology Obstetrics.
